# Patient-specific parameter estimates of glioblastoma multiforme growth dynamics from a model with explicit birth and death rates

**DOI:** 10.3934/mbe.2019265

**Published:** 2019-06-11

**Authors:** Lifeng Han, Steffen Eikenberry, Changhan He, Lauren Johnson, Mark C. Preul, Eric J. Kostelich, Yang Kuang

**Affiliations:** 1 School of Mathematical and Statistical Sciences, Arizona State University, Tempe, AZ 85287, USA; 2 Department of Neurosurgery, Barrow Neurological Institute, St. Josephs Hospital and Medical Center, Phoenix, AZ 85013, USA

**Keywords:** glioblastoma multiforme, parameter estimation, reaction-diffusion models, patient-specific models

## Abstract

Glioblastoma multiforme (GBM) is an aggressive primary brain cancer with a grim prognosis. Its morphology is heterogeneous, but prototypically consists of an inner, largely necrotic core surrounded by an outer, contrast-enhancing rim, and often extensive tumor-associated edema beyond. This structure is usually demonstrated by magnetic resonance imaging (MRI). To help relate the three highly idealized components of GBMs (i.e., necrotic core, enhancing rim, and maximum edema extent) to the underlying growth “laws,” a mathematical model of GBM growth with explicit motility, birth, and death processes is proposed. This model generates a traveling-wave solution that mimics tumor progression. We develop several novel methods to approximate key characteristics of the wave profile, which can be compared with MRI data. Several simplified forms of growth and death terms and their parameter identifiability are studied. We use several test cases of MRI data of GBM patients to yield personalized parameterizations of the model, and the biological and clinical implications are discussed.

## 1. Introduction

Glioblastoma multiforme (GBM) is a highly aggressive primary brain cancer, with median survival time from diagnosis on the order of 15 months; long-term survival is extremely rare [1]. Such rapid progression is promoted by highly proliferative and diffusely invasive cancer cells, which makes complete surgical removal impossible. Magnetic resonance imaging (MRI) is conventionally used to identify the location and characteristics of the tumor pre-operatively, to guide surgery, and to monitor and track progression and treatment response. Perioperatively, MRI is used to guide the resection of the tumor mass, to assess post-operatively the volume of tumor resected, and to target other adjunct treatment such as radiation therapy.

GBMs morphologically typically appear (at least at initial diagnosis) as roughly spherical but highly heterogeneous masses that often exhibit a (crudely speaking) three-layer structure. Within the tumor there is usually extensive cell necrosis, often accompanied by tumor cells, and a cystic component as well. An outer region, which typically appears as contrast-enhancing on T1-weighted gadolinium contrast-enhanced MRI, is cytologically typified by proliferating cells that then infiltrate into surrounding brain tissue. The surrounding brain tissue is generally seen to be edematous on T2-weighted or T2-FLAIR MRI and at surgery, due to vasogenic edema. Prior statistical analyses have found that edema is a prognostic indicator of patient survival [2], but the relationship is complex and appears to be mediated by the expression of vascular endothelial growth factor and the activity of related angiogenic genes [3] and various autocrine factors [4].

The standard of care for GBM patients was largely established by the 2005 clinical trial by Stupp *et al.* [5]. It comprises maximal surgical resection of the primary tumor, followed by six weeks of radiation to the gross tumor volume, plus a 2–3 cm margin, with concomitant oral temozolamide (TMZ), and 6–12 months of maintenance TMZ chemotherapy [6]. Maximal surgical resection appears to offer some survival benefit. Nevertheless, the absolute survival benefit of even the most effective therapy is typically on the order of months. The highly infiltrative nature of GBMs makes recurrence nearly inevitable, even with maximal resection and aggressive adjuvant therapy, although individual tumors vary in their degree of invasiveness.

Given the grim situation, mathematical modeling has been proposed as a method to better understand the biophysical rules underlying GBM growth, with the ultimate goal to provide more effective therapy. Mathematical models have been widely applied to a variety of cancers and to cancer treatment in general [7], and GBM is the focus of many such works (see [8] for a review). A popular class of cancer models takes the form of a system of reaction-diffusion equations. In many cases [9, 10, 11], such systems generate a traveling-wave solution, with the traveling-wave speed of great interest, as it is an indicator of how fast the cancer progresses.

Variants of the Fisher-Kolmogorov equation [12], originally introduced in the 1930s, were first suggested (to our knowledge) as models for GBM growth by J. D. Murray and coworkers. The Fisher-Kolmogorov model is given by

(1.1) $$\frac{\partial c}{\partial t}=\nabla \cdot (D\nabla c)+\rho c\left(1-\frac{c}{K}\right),$$

where *c*(*x*, *t*) is the cancer cell density at location *x* and time *t*, *D* is a diffusion coefficient, *ρ* is the intrinsic tumor cell growth rate, and *K* is the local carrying capacity. (Variants include a linear version that replaces the logistic growth term with a simple exponential growth rate, *ρc*.) Murray and coworkers have used them to explore the effect of chemotherapy [13], to quantify patients’ survival as a function of the extent of surgical resection [14], and to estimate the time of tumor initiation [15].

Other authors have suggested that the net growth and diffusion parameters of model (1.1) may be estimated by image differencing when two sequential, pre-treatment patient MR series are available [16, 17, 18]. Such a procedure is problematic, however, because changes in the tumor in images taken a few days or weeks apart tend to be small and are convolved with image co-registration errors [19]. Some patients may be treated with steroids following initial diagnosis to reduce tumor-related edema and resulting neurological symptoms, which may alter the brain geometry and imaging appearance of the tumor at subsequent times [20, 21].

Model (1.1) can yield a dense tumor core with an advancing front, but it cannot capture the heterogeneity between live and necrotic tumor cells, as it assumes that all cells are equally viable. While several modeling efforts have taken into account various proliferating, migrating, and necrotic cell components (e.g., [22, 23]), they are too complicated to be reliably parameterized by the limited number of patient MRI series in typical clinical cases. The motivation for this work is to extend model (1.1) to include necrotic cells in a simplified way, such that patient-specific model parameters can be estimated from suitable measurements of MR images acquired at a single time point.

T1-weighted MRI sequences of GBM often show a partially necrotic core surrounded by a bright enhancing rim that correlates with high blood vessel density and, presumably, with rapid cell proliferation. Neurosurgical and biopsy studies indicate that this core and rim are usually surrounded by a large expanse of edema, which is best visualized on T2-weighted MRI and has been found to correspond with a component of diffusely invasive GBM cells [24]. By approximating the tumor as a sphere, we may be able to identify three *idealized* digital marks from imaging: necrotic radius, enhancing radius, and what we shall call the “T2” or “maximum” radius. We hypothesize that a relatively simple mathematical model framework can capture all these three digital marks and yield insights into the relative contributions of cellular proliferation, motility, and necrosis to the observed image features.

The next section describes our model and its assumptions. We demonstrate that the model has a traveling-wave solution and present the approximate wave profile. We describe a simple procedure to estimate patient-specific parameters by fitting the approximate wave profile to a tumor profile derived from patient MRIs. The identifiability of the model parameters is also discussed. We apply this parameter estimation procedure to obtain the key model parameters (consisting of the rate of cancer cell proliferation, death, and diffusion) for several patients.

## 2. Model and method

### 2.1. Model description

Our proposed model of the growth of GBM is a system of reaction-diffusion equations:

(2.1a) $$\frac{\partial p}{\partial t}=\nabla \cdot \left[\left(\frac{Dp}{p+q}\right)\nabla (p+q)\right]+\tilde{g}(w)p-\tilde{\delta }(w)p,$$

(2.1b) $$\frac{\partial q}{\partial t}=\nabla \cdot \left[\left(\frac{Dq}{p+q}\right)\nabla (p+q)\right]+\tilde{\delta }(w)p,$$

where

(2.2) w=1−p−q,

and *p*(*x*, *t*) and *q*(*x*, *t*) represent the proliferating and quiescent cell densities at time *t* and location *x*, respectively; quiescent cells are functionally equivalent to necrotic cells in this framework. We assume that the flux of total population due to migration is −*D*∇(*p* + *q*), where *D* is a constant diffusion coefficient. It is further assumed that the proportion of the total flux contributed by each cell type equals its proportion of the total population. This form of diffusion was used in [25] to account for the key property of contact inhibition in cancer cell movement, with the underlying assumption that the two cell populations move together with equal motility, unaffected by necrotic cells. This type of model has successfully captured the structure of a growing tumor.

The per capita birth rate is $\tilde{g}(w)$; proliferating cells become quiescent at the per capita rate $\tilde{\delta }(w)$, where *w* (Eq. 2.2) represents the availability of space or some generic nutrient, which we will call *growth factor* henceforth. We have scaled the maximum cell density to be 1. In our model, necrosis is not explicitly included but can be regarded as being lumped into *q*. Insofar as quiescent cells cannot become proliferative, $\tilde{\delta }(w)$ can be viewed as a functional death rate. Our motivation in keeping the model framework relatively simple is to be able to estimate model parameters directly from clinical MRI imaging that is sparse in time.

To make the model biologically reasonable, we impose the following constraints on *g*(*w*) and *δ*(*w*):

(2.3) $${\tilde{g}}^{\prime }(w)\geq 0,\;{\tilde{\delta }}^{\prime }(w)\leq 0,\;\tilde{g}(1)\geq \tilde{\delta }(1)=0,\;\tilde{\delta }(0)>\tilde{g}(0)=0.$$

That is, birth (death) should increase (decrease) with the availability of the growth factor; there is more birth than death at maximum values of the growth factor; and there is only death with no growth in the absence of growth factor. It is also assumed that the death rate is negligible at maximum values of the growth factor. With these assumptions, we observe numerically that with suitable initial conditions, the solution of (2.1) stays positive and is bounded (*p* + *q* ≤ 1) for all *t*.

We can estimate only up to three parameters based on the necrotic, enhancing, and maximum radii to be measured from MRI images. Therefore, we place a few more restrictions on $\tilde{g}(w)$ and $\tilde{\delta }(w)$ to simplify the estimation of model parameters and to ensure their identifiability. We assume that the proliferation rate at maximum growth factor is *ρ* and that the death rate at zero growth factor is *k* and incorporate these parameters into $\tilde{g}$ and $\tilde{\delta }$, respectively; that is, $\tilde{g}(w=1;\rho )=\rho$ and $\tilde{\delta }(w=0;k)=k$. For reasons that will become clear later, we pick a functional form that can be written as $\tilde{g}(w;\rho )=\rho g(w)$ and $\tilde{\delta }(w;k)=k\delta (w)$. Some examples include the cumulative distribution function of the beta distribution family (cf. the left pane of Figure 4). These additional assumptions impose little impact on the generality of our model. The benefit of including them will become clear in Section 2.3.

### 2.2. Approximate wave profile

In most biological applications of reaction-diffusion models, solutions take the form of traveling waves. MRI images of GBM cancer growth suggest that we can approximate the evolution of the tumor by a traveling-wave solution of its growth model. To uniquely identify and accurately approximate GBM growth model parameters, it is highly desirable to obtain some analytic approximation of the traveling wave, to enable computational matching of the image wave profile and the approximate model wave profile. For this purpose, we consider one spatial dimension, which suffices insofar as the tumor is approximately spherical, and, at the time of diagnosis, its radius is large enough so that radial effects are negligible. With these assumptions, model (2.1) takes the form

(2.4a) $$\frac{\partial p}{\partial t}=\frac{\partial }{\partial x}\left[\left(\frac{Dp}{p+q}\right)\frac{\partial }{\partial x}(p+q)\right]+\rho g(w)p-k\delta (w)p$$

(2.4b) $$\frac{\partial q}{\partial t}=\frac{\partial }{\partial x}\left[\left(\frac{Dq}{p+q}\right)\frac{\partial }{\partial x}(p+q)\right]+k\delta (w)p.$$

We nondimensionlize the system using the characteristic length $\sqrt{D/k}$ and the characteristic time 1/*k* so that $x=\sqrt{D/k}\hat{x}$ and $t=\hat{t}/k$, which leads to

(2.5a) $$\frac{\partial p}{\partial \hat{t}}=\frac{\partial }{\partial \hat{x}}\left[\left(\frac{p}{p+q}\right)\frac{\partial }{\partial \hat{x}}(p+q)\right]+\hat{\rho }g(w)p-\delta (w)p$$

(2.5b) $$\frac{\partial q}{\partial \hat{t}}=\frac{\partial }{\partial \hat{x}}\left[\left(\frac{q}{p+q}\right)\frac{\partial }{\partial \hat{x}}(p+q)\right]+\delta (w)p,$$

where $\hat{\rho }=\rho /k$. We seek a traveling wave solution of the form $p(\xi )=p(\hat{x}-c\hat{t})$, $q(\xi )=q(\hat{x}-c\hat{t})$, where *c* is the wave speed. Substituting these into (2.5) gives

(2.6a) $$\frac{d}{d\xi }\left[\left(\frac{p}{p+q}\right)\frac{d}{d\xi }(p+q)\right]+c\frac{dp}{d\xi }+\hat{\rho }g(w)p-\delta (w)p=0$$

(2.6b) $$\frac{d}{d\xi }\left[\left(\frac{q}{p+q}\right)\frac{d}{d\xi }(p+q)\right]+c\frac{dq}{d\xi }+\delta (w)p=0.$$

Linearizing at the wave head, i.e., substituting the ansatz *p* = *Ae*^−*r*ξ^ and *q* = *Be*^−*r*ξ^ into (2.6), gives (*r*^2^ − *cr* + *ρ*)*A* = 0. For a biologically realistic wave front, we expect *A* > 0, *B* > 0, and *r* > 0. This requires that *c*^2^ > 4*ρ*, which implies that the minimum speed of the wave is $c_{\text{min}}=2\sqrt{\hat{\rho }}$. It is numerically verified that the minimum speed is exactly the asymptotic speed, i.e., *c* = *c*_min_.

To obtain an approximate wave profile, we adopt a method first used by Canosa [26]. We rescale the wave coordinate as *z* = −ξ/*c*, which leads to

(2.7a) $$\frac{1}{c^{2}}\frac{d}{dz}\left[\left(\frac{p}{p+q}\right)\frac{d}{dz}(p+q)\right]-\frac{dp}{dz}+\hat{\rho }g(w)p-\delta (w)p=0,$$

(2.7b) $$\frac{1}{c^{2}}\frac{d}{dz}\left[\left(\frac{q}{p+q}\right)\frac{d}{dz}(p+q)\right]-\frac{dq}{dz}+\delta (w)p=0.$$

**Figure 1.**
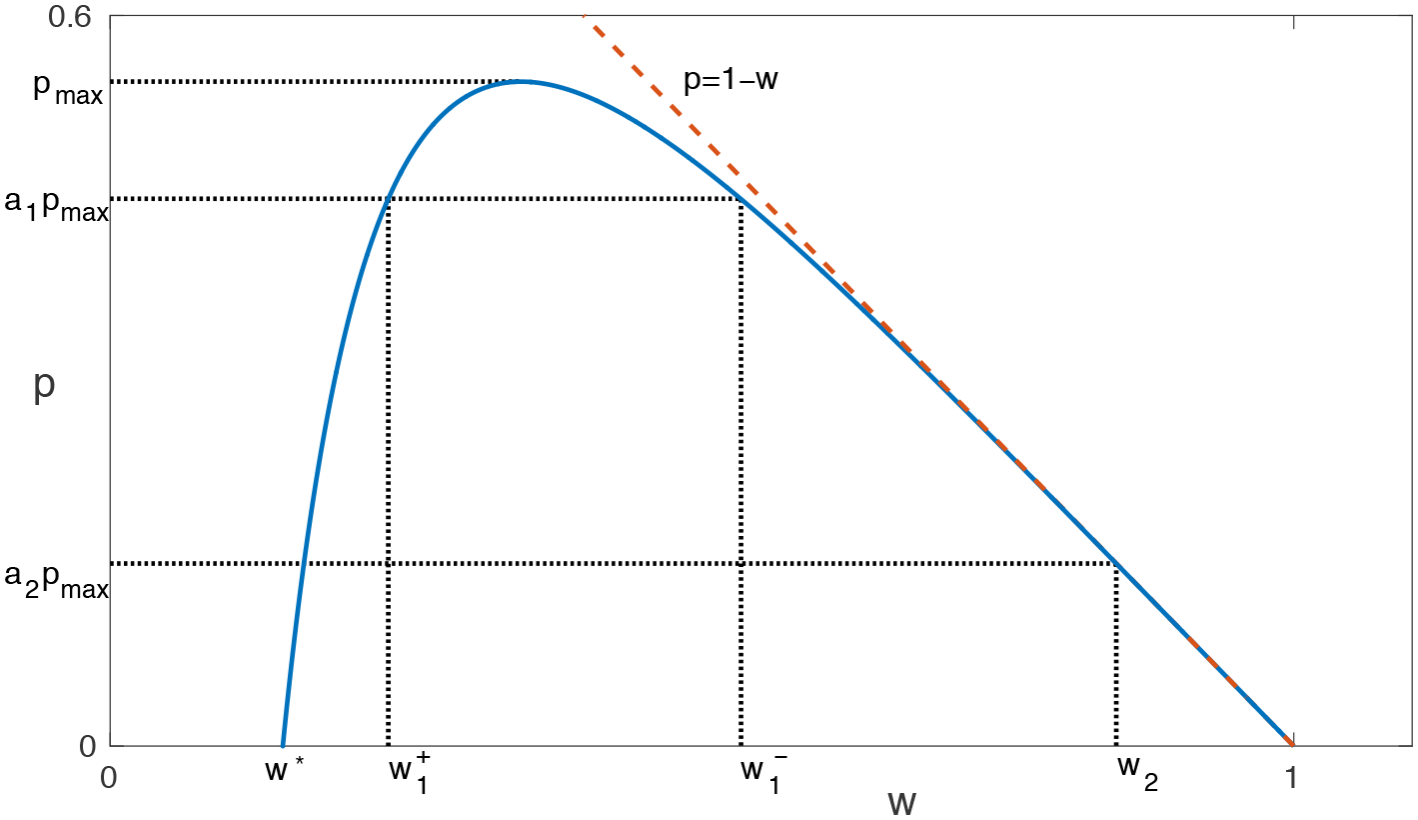
A typical trajectory that connects (0, 1) and (0, w*) in the phase plane. Given *δ*(*w*) and *g*(*w*), this trajectory can be found by integrating (2.9). It represents an approximate traveling-wave solution. See the appendix for a proof of its existence under general assumptions.

Assuming that 1/*c*^2^ is small, we neglect each first term of (2.7). Writing the resulting system in terms of *p* and *w*, we obtain the reduced system

(2.8a) $$\frac{dp}{dz}=p(\hat{\rho }g(w)-\delta (w)),$$

(2.8b) $$\frac{dw}{dz}=-p\hat{\rho }g(w),$$

which is amenable to phase-plane analysis. The approximate wave solution corresponds to a trajectory that leaves (0, 1) and ends at (0, w*), with w* ∈ [0, 1) (see Figure 1). (In the appendix, we show that such a trajectory exists, given the assumptions (2.3).) Dividing (2.8a) by (2.8b) yields

(2.9) $$\frac{dp}{dw}=\frac{\delta (w)}{\hat{\rho }g(w)}-1.$$

Upon integration, we obtain *p* as a function of *w*, which we will use in the next section.

### 2.3. Parameter estimation

**Figure 2.**
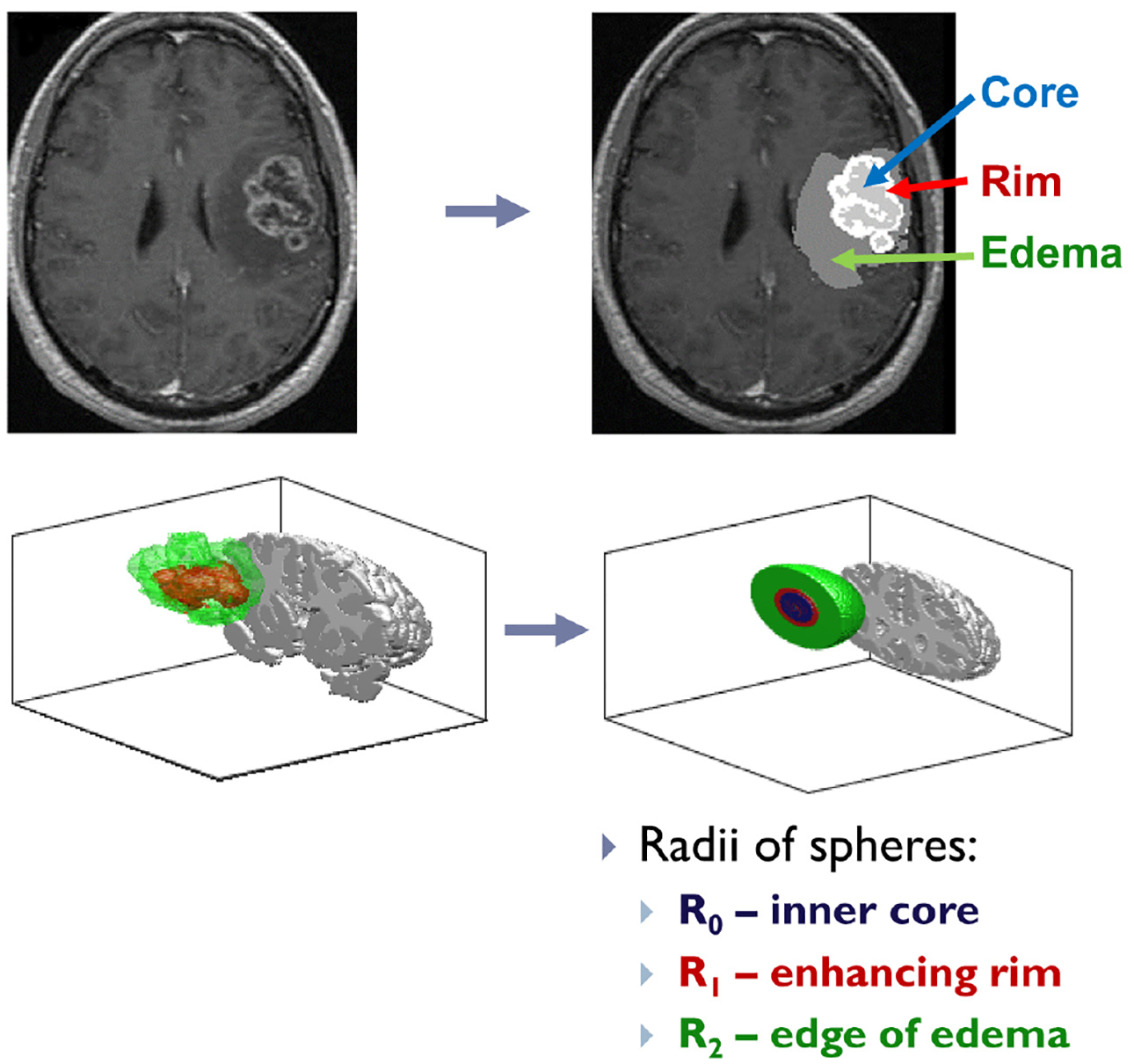
The top half of the figure shows an example patient MRI registered to the standard brain domain, with the three tumor segments, necrotic core, enhancing rim, and tumor-associated edema highlighted on a single 2-D slice. The full 3-D segmentation, and the equivalent tumor sphere with associated radii, *R*_0_, *R*_1_, and *R*_2_, is shown in the lower half.

**Figure 3.**
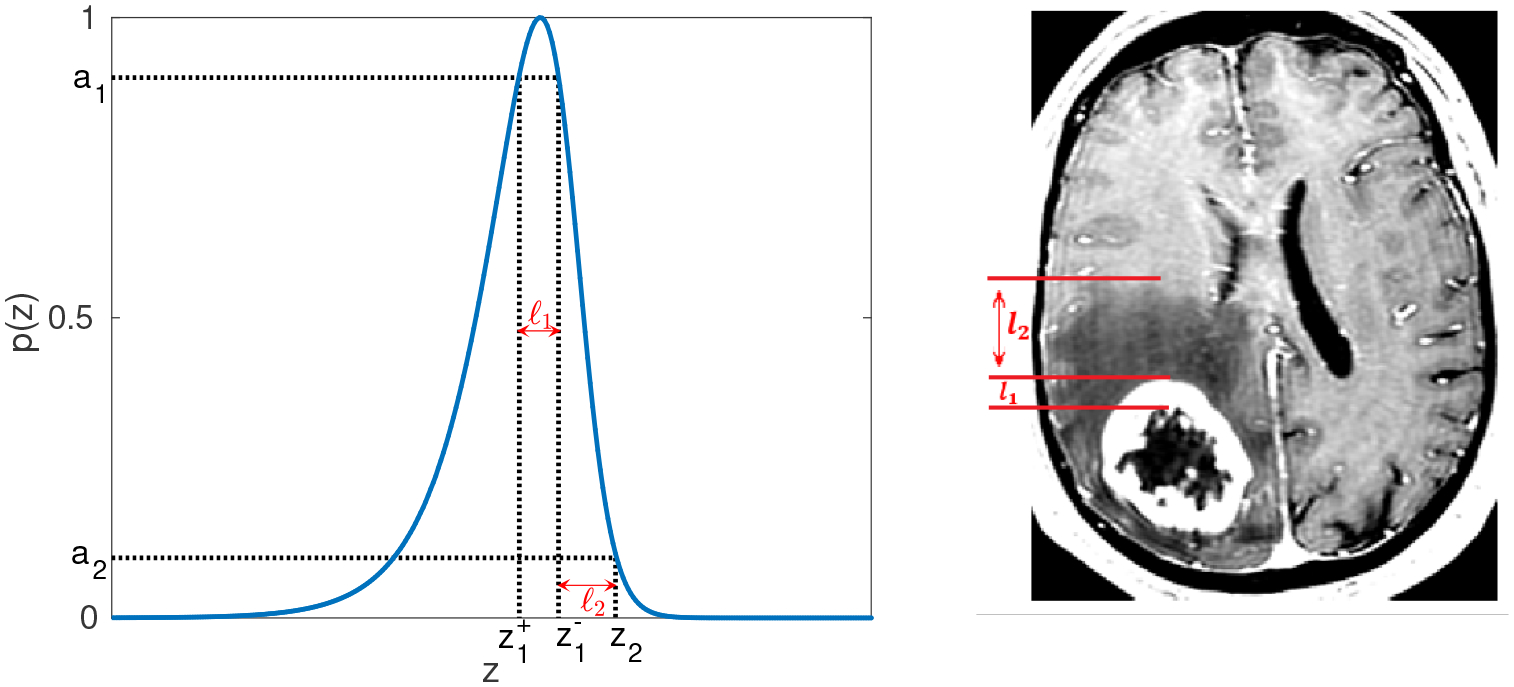
Left: normalized wave profile generated by the model in the *z* coordinate. Right: tumor profile seen in MR image. Parameter estimation is done by matching model-derived quantities, e.g., *ℓ*_1_ and *ℓ*_2_, to the corresponding image-derived ones.

From clinical MRI data, we may derive three idealized radii: *R*_0_, *R*_1_, and *R*_2_, representing respectively the radius of the inner necrotic core, the radius to the edge of the contrast-enhancing rim, and the radius to the outer edge of tumor-associated edema. Such data have been extracted from a series of anonymized patient MRI data consisting of T1-contrast enhanced and T2-weighted MRIs at initial diagnosis. Using the publicly available MATLAB software package, Statistical Parametric Mapping 12 (SPM 12) [27], MRIs are initially registered to a standard brain space, and then, using Slicer 3D [28] software, the total necrotic core volumes, enhancing rim volumes, and tumor-associated edema volumes are determined from semi-manual tumor segmentation. Finally, these volumes are converted to radii assuming a spherical tumor geometry. The width of the proliferating rim, denoted as *L*_1_, and the width of the edematous rim, denoted as *L*_2_, can be calculated as *L*_1_ = *R*_1_ − *R*_0_ and *L*_2_ = *R*_2_ − *R*_1_, as demonstrated visually in Figures 2 and 3.

We assume that contrast-enhancing regions of T1-weighted images correspond to high densities of proliferating tumor cells and that edematous regions on T2-weighted imaging correspond to low densities. We denote the respective detection thresholds for T1 and T2 imaging as *a*_1_*p*_max_ and *a*_2_*p*_max_, where 0 < *a*_2_ < *a*_1_ < 1 and *p*_max_ = max_*z*_ *p*(*z*), i.e., the maximum density of proliferating cells given by the traveling-wave solution.

Often only a single MRI series is available before surgery, although in some cases, a diagnostic MRI followed some days or weeks later by a pre-surgery MRI may be available. In the latter case, the image-derived wave velocity *V* is the change in tumor radius divided by the length of the time interval.

From our approximate wave profile, we can compute the corresponding quantities to match with MR images (cf. Figure 3). The wave-solution based approximation of the width of the proliferating rim, denoted *ℓ*_1_, and the width of the edematous rim, denoted *ℓ*_2_, (in dimensional form) are computed as

(2.10a) $$l_{1}=\frac{2\sqrt{D\rho }}{k}\int_{w_{1}^{-}}^{w_{1}^{+}}\frac{dz}{dw}dw,$$

(2.10b) $$l_{2}=\frac{2\sqrt{D\rho }}{k}\int_{w_{2}}^{w_{1}^{-}}\frac{dz}{dw}dw,$$

respectively, where $w_{1}^{\pm }$ and *w*_2_ satisfy, respectively, $p\left(w_{1}^{\pm }\right)=a_{1}p_{\text{max}}$ and *p*(*w*_2_) = *a*_2_*p*_max_. Here *p*(*w*) is obtained by integrating Eq. (2.9) (see appendix for details). Additionally, the model-derived wave speed $c=2\sqrt{\rho D}$ can be matched with the image-derived speed *V*. Thus we have three nonlinear equations

(2.11) $$l_{1}=L_{1},\;l_{2}=L_{2},\;c=V,$$

from which we hope to find the parameters *D*, *ρ*, and *k*. Given our assumptions, we can simply take the ratio of (2.10a) and (2.10b), which gives

(2.12) $$f(\hat{\rho })\equiv \frac{\int_{w_{1}^{-}}^{w_{1}^{+}}\frac{dz}{dw}dw}{\int_{w_{2}}^{w_{1}^{-}}\frac{dz}{dw}dw}=\frac{L_{1}}{L_{2}},$$

insofar as the integrals are functions of $\hat{\rho }$. Equation (2.12) can be solved for $\hat{\rho }$ analytically in special cases or numerically in general. The monotonicity of $f(\hat{\rho })$ is important for the identifiability of parameters. Once we find $\hat{\rho }$, i.e., the ratio *ρ*/*k*, all parameters can be found by back substitution.

The above method requires two MR scans taken at two consecutive times prior to surgery to obtain an image-derived estimate of wave speed. If no second MR series is available, then tumor age may be estimated by the tumor radius divided by the wave speed. However, the estimate depends on which radius (*R*_1_ or *R*_2_) is used, because the tumor grows exponentially at first and linearly later on [7]. This initial exponential growth stage needs to be taken into account as a correction to the aforementioned tumor age estimation. Suppose that for 0 ≤ *t* ≤ t*, quiescence is negligible and the proliferating cancer cells grow exponentially from a point source of density *p*_0_, and that for *t* > t*, the tumor grows as a traveling wave with speed $2\sqrt{\rho D}$. By equating the two age estimates, we obtain

(2.13) $$\frac{R_{1}-R_{1}^{*}}{2\sqrt{\rho D}}=\frac{R_{2}-R_{2}^{*}}{2\sqrt{\rho D}},$$

where

(2.14) $$R_{i}^{*}=t^{*}\sqrt{4D\rho -\frac{4D}{t^{*}}\text{ln}\left(\frac{a_{i}{\left(4\pi Dt^{*}\right)}^{3/2}}{p_{0}}\right)},\;i=1,2,$$

where *R*_1_ and *R*_2_ are respectively the T1 and T2 radii at *t* = t* (see details of $R_{1}^{*}$ and $R_{2}^{*}$ in the appendix).

Replacing the last equation in (2.11) with (2.13), we again have three equations. To solve them for the unknown parameters, we first take (2.10a) over (2.10b) as before to obtain (2.12). It can then be solved for the ratio $\hat{\rho }=\rho /k$. Substituting this expression back to either *ℓ*_1_ = *L*_1_ or *ℓ*_2_ = *L*_2_ gives the raito *ρ*/*D*. Finally, expressing *D* and *k* in terms of *ρ*, (2.13) can be solved for *ρ*, and *D* and *k* follow.

## 3. Results

**Figure 4.**
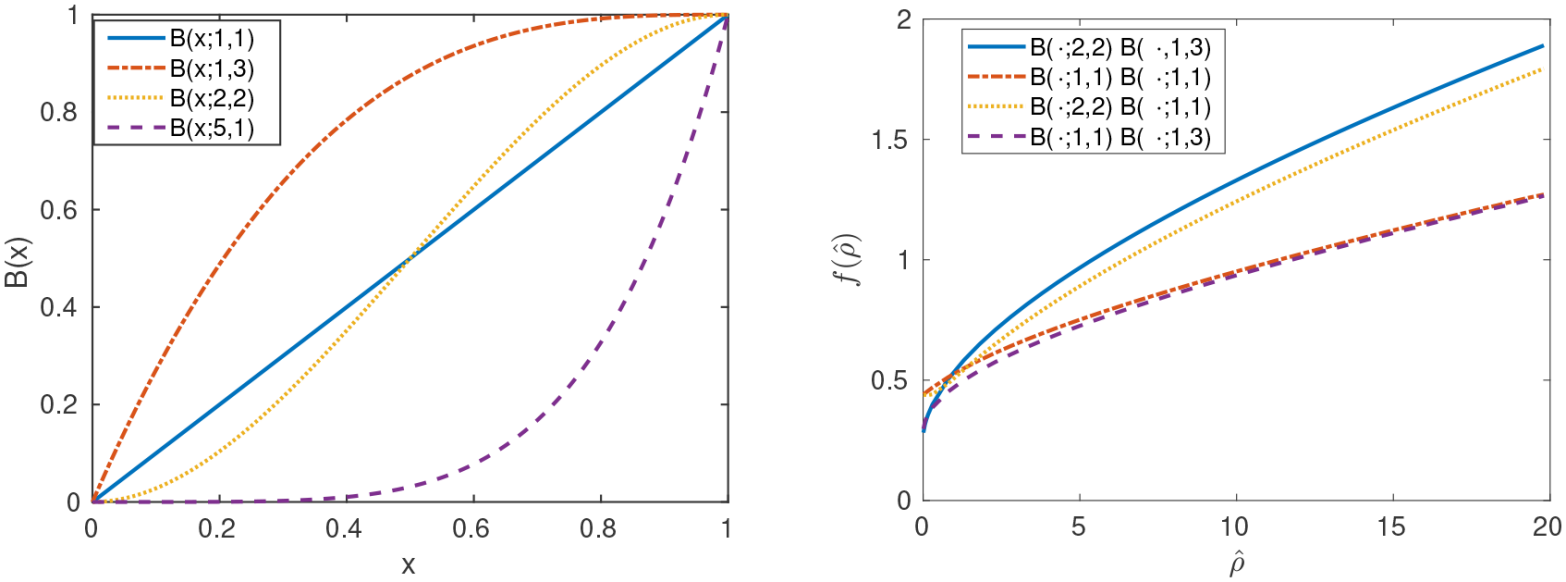
Left: Cumulative distribution functions of some beta distributions. These functions satisfy Eq. (2.3) and serve as candidates to represent biological response to limitation of growth factors. Right: monotonicity of $f(\hat{\rho })$ given different choices of *g*(*w*) and *δ*(*w*) as indicated in the legend. All choices lead to a monotonic function $f(\hat{\rho })$ and hence identifiable parameters.

Monotonicity is crucial for parameter identifiability, so we first investigate the monotonicity of *f* for some specific choices of *g*(*w*) and *δ*(*w*). Given the restrictions described in Section 2.1, the cumulative density function (CDF) of the Beta distribution family suits our purposes. Therefore, we let *g*(*w*) = *B*(*w*; *α*_*g*_,*β*_*g*_) and *δ*(*w*) = 1 − *B*(*w*; *α*_*δ*_, *β*_*δ*_), where *B*(*w*; *α*, *β*) is the CDF of the beta distribution with shape parameters *α* and *β*. By varying *α* and *β*, we can get linear, sigmoidal, and concave up/down curves (see the left pane of Figure 4). Our framework is robust to those choices, that is, the monotonicity of $f(\hat{\rho })$, defined by Eq. (2.12), is preserved (right pane of Figure 4). Sigmoidal-shaped growth and death functions (*g* and *δ*, respectively) may provide biologically realistic response functions to limited growth factors (most enzymatic reaction rates have sigmoidal shapes with respect to reactant concentration). Given this family of functions, we now consider the question of estimating patient-specific tumor growth and death rates from MR imaging.

We parameterize our model with patient data in which there is only one MRI scan before surgery. In Table 1, we summarize the image-derived tumor radii and the corresponding parameters estimated by the method introduced in the previous section. The parameters *a*_1_ = 0.9 and *a*_2_ = 0.1 are adapted from values found in the literature [16], while *p*_0_ = 0.02 and t* = 60 days are hypothetical values. The parameters vary considerably among individual patients.

**Figure 5.**
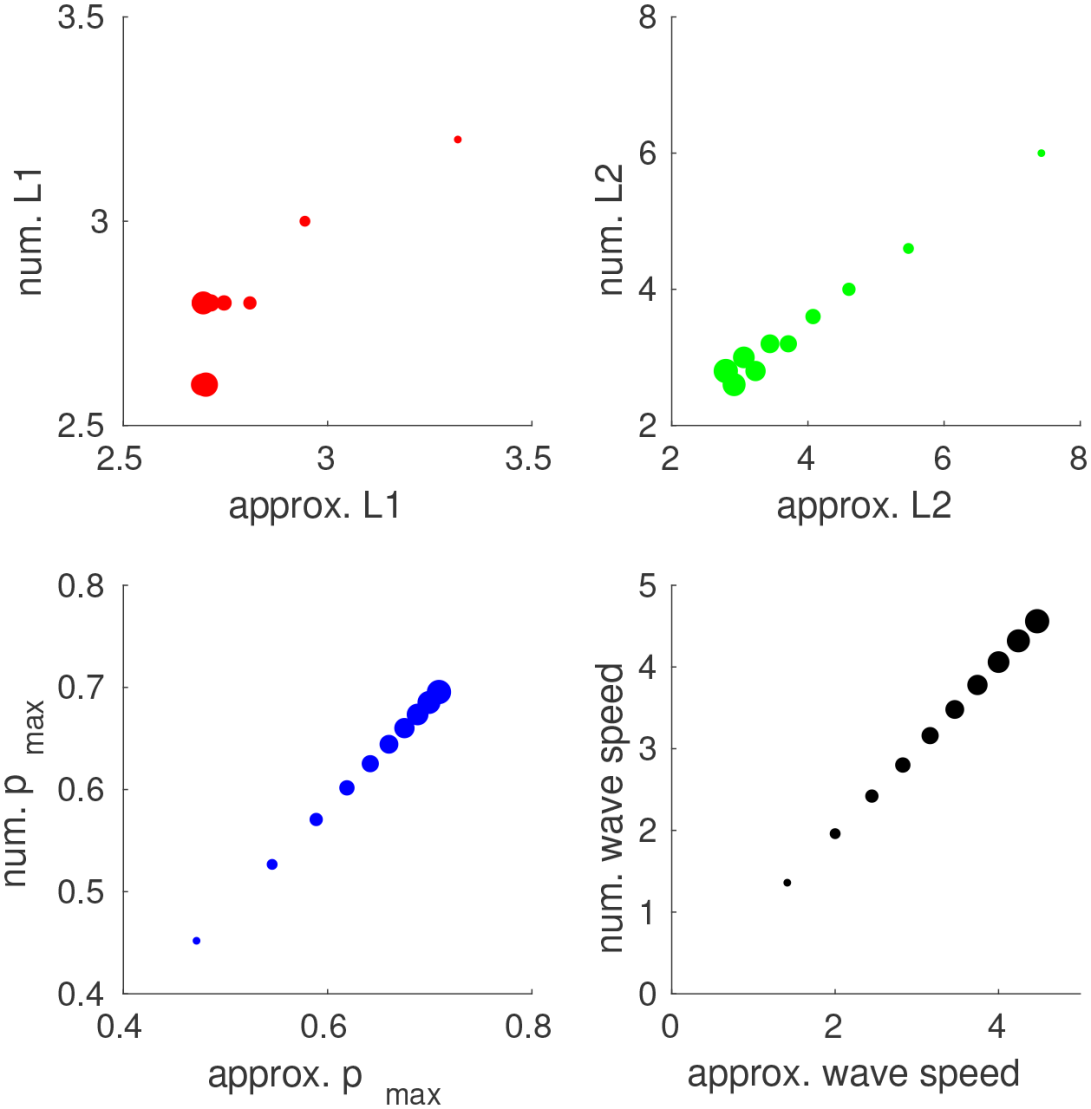
Scatter plots of approximate wave profile characteristics (on horizontal axis) verses the ones obtained by numerical simulation (on vertical axis) for a range of $\hat{\rho }$ from 0.5 to 5 by increments of 0.5. The size of the dot corresponds to the value of $\hat{\rho }$. The dots scatter closely to the diagonal line with slope 1, indicating agreement between the numerical solution and our approximation.

We compare our approximate quantities to those obtained from the numerical solution of the model. As shown in Figure 5, the approximated results match well with the numerical results except for some discrepancy for *L*_2_ when $\hat{\rho }$ is small. This result is not a surprise, because the approximation assumes that $c=2\sqrt{\hat{\rho }}$ is large. Moreover, the numerical approximation of *L*_2_ is prone to errors due to the fixed grid size and large rate of change around the threshold of *L*_2_. Overall, we believe that our approximation is accurate for the parameter ranges estimated from the image data.

## 4. Discussion

In this work, we have extended the Fisher-Kolmogorov reaction-diffusion model of GBM growth, Eq. (1.1), to explicitly separate the cancer cell birth and death (or quiescence) processes, which are described in terms of generic functions that depend upon an implicit nutrient or growth factor. We specify the birth and death processes, *g*(*w*) and *δ*(*w*), respectively, by the cumulative distribution function of a beta distribution, each uniquely specified by a single parameter, *ρ* and *k*. Thus, along with the diffusion coefficient, *D*, our model describes cancer growth via three parameters, *D*, *ρ*, and *k*, and yields a tumor morphology (in one dimension) consisting of a necrotic core, a high-density rim, and an outer low density rim, which we may correlate to three radii, *R*_0_, *R*_1_, and *R*_2_, that can be estimated from a single patient MR image.

We have demonstrated that our reaction-diffusion system has a traveling-wave solution, which is common in such systems. Studies on this topic date back to the Fisher’s work in the 1930s on the spread of advantageous genes [12]. Rigorous proof of the existence of a traveling-wave solution in a reaction-diffusion system often leads to phase-space analysis such as the one on the diffusive Lotka-Volterra equations [29]. Although in general a rigorous proof of a traveling-wave solution is a daunting task, our reduced system is amenable to phase-plane analysis, and the orbit that represents the traveling-wave solution can be identified (see appendix).

Via traveling-wave analysis, we have developed a method to estimate *D*, *ρ*, and *k* from as few as a single magnetic resonance image, based on certain growth assumptions. We have estimated these parameters for six patient test cases, as shown in Table 1. Because of the sparsity of imaging data for a typical patient, parameter identifiability in this case is provided by the monotonicity of the function $f(\hat{\rho })$ (as seen in left pane of Figure 4); our approach differs from more common statistical practices [30] that are appropriate when more data are available.

Disaggregating the net cell proliferation into birth and death processes not only aids in relating (simplified) tumor appearance on MRI to the model parameters, but it may provide useful valuable information for personalized treatment design, insofar as chemotherapy and radiotherapy target proliferating cells. Moreover, the structural information is also potentially useful, as drug dosages might be selected to ensure penetration through the width of the proliferating rim. Research along this general line has been conducted using model (1.1) [31].

Our analysis uses a more complex description of motility than simple diffusion. The diffusion term in Eq. (2.4) belongs to a more general category called *cross diffusion* [32]. It represents the phenomenon in which the gradient in the concentration of one species causes a flux of another species. The type of cross diffusion considered in this paper has been studied in a more general and theoretical context [33]. In a modeling study of avascular tumour growth [25], the authors justified the adoption of a proportion-based cross diffusion in a tumor-growth model by recognizing that tumor cell migration is “contact inhibited”: the presence of one type of cell halts the movement of the other. This type of cross diffusion can cause the solution to become negative [32], but we have not encounted this difficulty so far in our numerical simulations. We conjecture that, for suitable initial conditions, solutions remain positive, which will be a topic for future study. Other types of density-dependent diffusion have been considered in modeling GBM migration [34].

Despite the existence of many possible diffusion terms, the exact form of diffusion does not matter in the one-dimensional analysis, because the second derivatives are dropped in model (2.7). However, The diffusion coefficient does play an important role in the linearized wave head, where it affects the wave speed, and in the characteristic length where its square root scales the space. The scale-invariant part of the wave profile is mostly determined by the exact forms of the birth and death functions.

The major contribution of this paper is two novel methods to make patient-specific estimates of a three-parameter model of GBM grwoth from the limited MRI data that is typically available in clinical settings. It is possible to estimate the model parameters from a single pre-surgery image. Improved estimates may be possible when images are acquired at multiple time points.

As cancers progress, the underlying parameters describing their growth are unlikely to be static. Data assimilation refers to basic method of updating parameters as more data is acquired, and there has been research on applying full-fledged data assimilation to cancer modeling [35, 36]. The methods described in this paper can be incorporated as part of a future data assimilation system.

## Figures and Tables

**Table 1.** Radii of equivalent tumor sphere derived from T1 and T2 images and the corresponding vital parameters estimated by our protocol. We have preset *a*_1_ = 0.9, *a*_2_ = 0.1, *p*_0_ = 0.02 mm and t* = 60 days.

Patient	*R*_0_ (mm)	*R*_1_ (mm)	*R*_2_ (mm)	*D* (mm^2^day^−1^)	*ρ* (day^−1^)	*k* (day^−1^)
1	14.87	20.73	27.77	0.2852	0.2102	0.0602
2	20.48	26.34	38.24	1.2791	0.2624	0.3537
3	6.61	10.91	15.24	0.0825	0.1736	0.0327
4	22.87	26.96	37.03	0.9825	0.2590	0.7819
5	8.17	14.20	25.10	0.9769	0.2520	0.2260
6	8.29	15.83	20.35	0.0687	0.1652	0.0106

**Figure 6.**
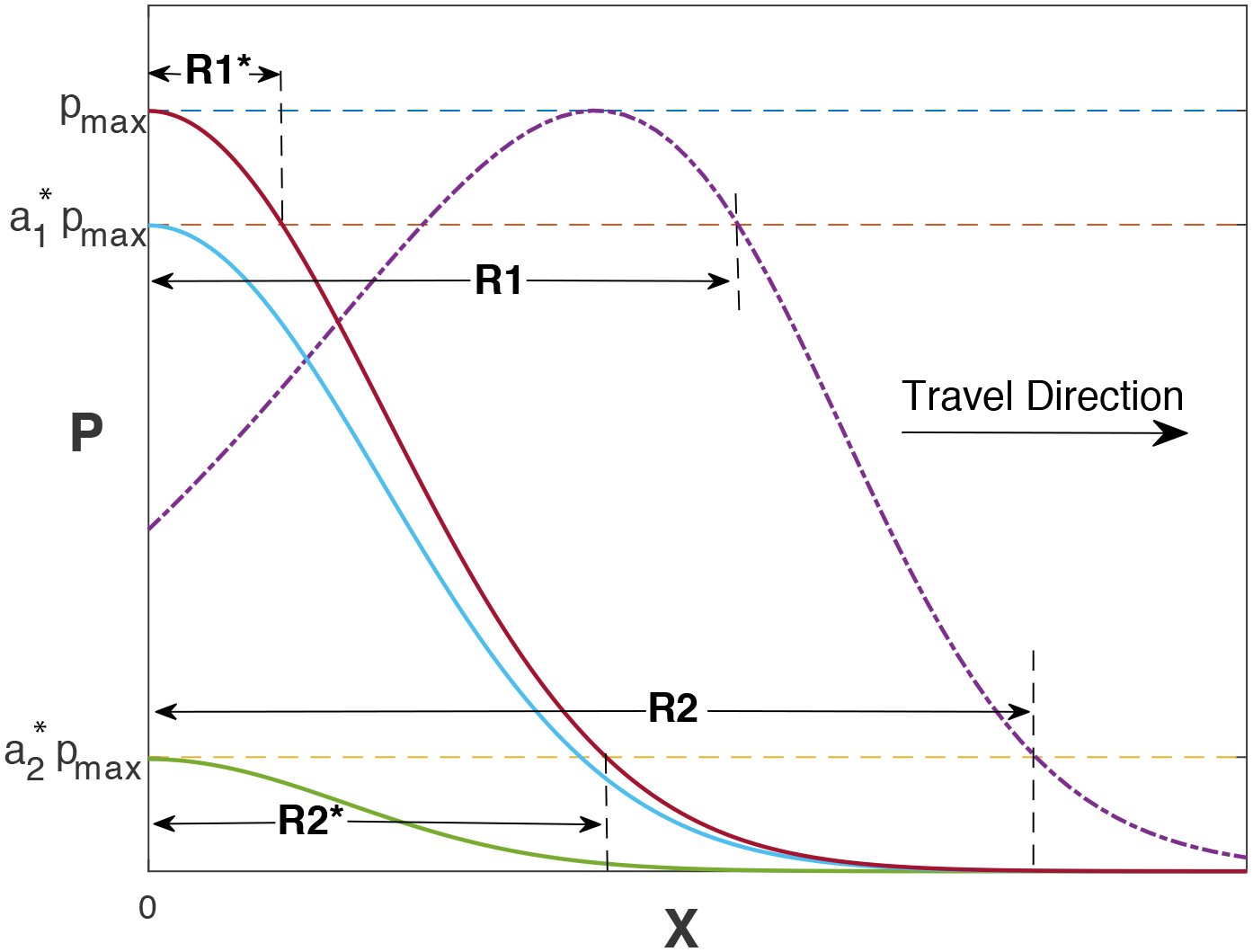
The transition of wave profile based on the two-stage tumor growth model. The dotted (purple) curve represents a stable wave profile generated by system (2.8). The solid curves are generated by Eq. (4.5) and represent the tumor’s exponential growth phase. The stable wave profile is formed after the exponential growth curve reaches *p*_max_ (red solid curve).

## Appendix

### Traveling-wave solutions

In the following, we rigorously establish the existence of traveling-wave solutions in system (2.8). We first show that the solutions of system (2.8) with positive initial values are non-negative and bounded.

**Lemma 1.** Assume that *g*(*w*) = *wG*(*w*), where *G*(*w*) is a bounded function. Then the solutions of system (2.8) with positive initial values are positive and bounded.

*Proof:* If *x*′ = *x f*(*t*, *x*) and *f* is a bounded function, then $x(t)=x\left(t_{0}\right)\text{exp}\left(\int_{t_{0}}^{t}f(s,x(s))ds\right)$, which is positive whenever *x*(*t*_0_) > 0. Since

(4.1) $$\frac{d(p+w)}{dz}=-\delta (w),$$

we see that (*p* + *w*) is bounded, which implies that both *p* and *w* are also bounded. □

For any *w** ∈ [0,1], the point (0, *w**) is an equilibrium of (2.8).

**Theorem 1.** System (2.8) admits positive traveling-wave solutions that correspond to heteroclinic orbits connecting the steady state (0, 1) to another steady state, (0, *w**).

*Proof:* We have performed a detailed phase-plane analysis of (2.8) to show the existence of a trajectory that starts from (1, 0) and ends at (*w**, 0), where *w** ∈ [0, 1). (See Figure 1.) First we notice that

(4.2) $$\frac{dp}{dw}=\frac{\delta (w)}{\hat{\rho }g(w)}-1,$$

(4.3) $$\frac{d^{2}p}{dw^{2}}=\frac{\delta^{\prime }(w)g(w)-\delta (w)g^{\prime }(w)}{\hat{\rho }g{\left(w\right)}^{2}},$$

Since *g*(*w*) and *δ*(*w*) are both positive functions and *g*′(*w*) > 0 and *δ*′(*w*) < 0 on *w* ∈ [0, 1], it follows that *d*^2^*p*/*dw*^2^ < 0 for all *w* ∈ [0, 1].

We show first that the trajectory starting from (1, 0) will never cross the line *p* = 1 − *w*. Because *g*(1) = 1, we have *δ*(1) = 0, and therefore, *dp*/*dw*|_*w*=1_ = −1. Since *d*^2^*p*/*dw*^2^ < 0, the slope of a trajectory with *w* < 1 will be greater than −1, which means it will not cross the line *p* = 1 − *w*. Also, because the solution components stay positive, the trajectory starting from (1, 0) will never cross the *p*-axis from right to left.

Because the functions *g* and *δ* are monotone, there is a unique value *w*^†^ ∈ (0, 1) such that $dp/{dw|}_{w=w^{†}}=0$. Moreover, $dp/{dw|}_{1>w>w^{†}}>0$ while $dp/{dw|}_{0<w<w^{†}}<0$.

Insofar as *w* is strictly decreasing and bounded from below by 0, there exists some *w** ∈ (0, 1) such that lim_*z*→∞_ *w*(*z*) = *w**. We claim that *w** < *w*^†^. Otherwise, *p* is a non-decreasing function, which implies that the trajectory approaches a positive steady state *E** = (*p**, *w**). However, the system (2.8) does not admit any positive steady states.

Let *a* ∈ (*w**, *w*^†^) and $b=\hat{\rho }g(a)-\delta (a)<0$. Since lim_*z*→∞_ *w*(*z*) = *w**, there is a *z** > 0 such that for *z* ≥ *z** and *w* < *a*. Therefore, for *z* ≥ *z**, *dp*/*dz* < *bp*, which implies that lim_*z*→∞_ *p*(*z*) = 0. Hence, system (2.8) admits positive traveling-wave solutions that correspond to heteroclinic orbits connecting the steady state (0, 1) to another steady state, (0, *w**). □

### Rim width

The trajectory in Figure 1 corresponds to a traveling-wave profile in the *z* coordinate as shown on the left pane of Figure 3. Because of our choice of nondimensionlization, $x=-(2\sqrt{D\rho }/k)z$, by definition we have the rim width in the dimensional form

(4.4) $$l_{1}=\frac{2\sqrt{D\rho }}{k}\left(z_{1}^{+}-z_{1}^{-}\right)=\frac{2\sqrt{D\rho }}{k}\int_{z_{1}^{-}}^{z_{1}^{+}}dz$$

where $p\left(z_{1}^{\pm }\right)=a_{1}p_{\text{max}}$. We have taken *p* as a function of *z* (cf. the left pane of Figure 3). We can also take *w* as a function of *z* to make a change of variable to the above integral, which gives Eq. (2.10a). A similar argument applies to (2.10b).

### Derivation of $R_{1}^{*}$ and $R_{2}^{*}$

Tumor growth can be separated into two stages. First, the tumor cells grow exponentially until the cell density is high enough (*p*_max_) to form a stable wave profile. Afterward, the growth of tumor cells is described by (2.8). The radii *R*_1_ and *R*_2_ are observed from clinical MRI data and correspond respectively to the distance from the center of the tumor to the edge of the enhancing rim and to the edge of the edematous region on T2 imaging; they correspond to tumor dynamics before the stable wave profile has formed (cf. Figure 6).

During the exponential growth phase, say from 0 < *t* < *t**, quiescence is negligible and the governing equation of tumor cell density is

(4.5) $$\frac{\partial p}{\partial t}=D\frac{1}{r^{2}}\frac{\partial }{\partial r}\left(r^{2}\frac{\partial p}{\partial r}\right)+\rho p,$$

where spherical symmetry is used, as we assume that the tumor is spherical when its radius is small. This linear equation has the Green’s function

(4.6) $$p(r,t)=\frac{1}{{\left(4\pi Dt\right)}^{3/2}}\text{exp}\left(\rho t-\frac{r^{2}}{4Dt}\right)$$

(see, e.g., [37] Page 314 and [38] Page 285). Suppose that the tumor starts at *t* = 0 as a point source with density *p*_0_. It follows that at *t* = *t**, the position of the tumor front at density *p* = *a*_*i*_ is given by Eq. (2.14).
